# Journalistic Consolidation

**Published:** 1878-08

**Authors:** 


### JOURNALISTIC CONSOLIDATION.

*The Cincinnati Lancet and Observer* and *The Cincinnati Clinic*, two of the most prominent medical journals of the

West, united their fortunes and destinies "for better or for worse," on the 6th of July.

The Lancet and Observer was established about forty years ago. It has always been in charge of an able management, and has uniformly had the best writers in the profession, in the West at least, for its contributors. It has exercised a great power for good in the profession, indeed it might rightly be considered one of the medical educators of the West. It was issued every month, and was constantly becoming more and more attractive in appearance and in the amount and character of its contents. Each number recently has been a volume of one hundred and ten pages.

The Clinic was established seven years ago, and had attained a good and solid footing in all the essentials for a permanent enterprise. This was issued each week and was less voluminous than the Lancet, but nevertheless it was always filled with choice and fresh matter.

In this union there will be the solidity, age and dignity of the Lancet with the sprightliness, energy and frequent issue of the Clinic, by which, without doubt we will have one of the best medical journals in this country.

This journal in its present form will be an invaluable one to the dentist. Almost everything on its pages would repay reading, and much of it close study, and doubtless the editors would be pleased to receive and publish contributions on various subjects from the dentist's standpoint. Let somebody make the experiment.

We advise every reader of the REGISTER to subscribe for the Lancet and Observer.

